# Management of immune-mediated glomerular diseases in the elderly

**DOI:** 10.1080/0886022X.2024.2411848

**Published:** 2024-10-08

**Authors:** Andrea Angioi, Wisit Cheungpasitporn, Nicola Lepori

**Affiliations:** a S.C.D.U. Nefrologia, Dialisi e Trapianto, ARNAS Brotzu, Cagliari, Italy; b Division of Nephrology and Hypertension, Mayo Clinic, Rochester, Minnesota, USA; c Dipartimento di Scienze Mediche e Sanità Pubblica, Università di Cagliari, Cagliari, Italy

**Keywords:** Glomerulonephritis, elderly, age, kidney biopsy, immunosuppression

## Abstract

The management of immune-mediated nephropathies in the elderly presents unique challenges due to age-related physiological changes, comorbidities, and frailty. This review addresses the clinical workup, diagnostic evaluation, and treatment strategies for this rapidly growing patient population. We highlight the inadequacies of current classification systems and the lack of evidence-based guidelines tailored to individuals ≥75 years. The review discusses the specific considerations in diagnosing and treating common conditions such as minimal change disease, focal and segmental glomerulosclerosis, membranous nephropathy, ANCA-associated vasculitis, infection-related and post-infectious glomerulonephritis, and anti-GBM disease. Managing these diseases requires a nuanced approach due to age-related changes in the immune system and the presence of multiple comorbidities. Immunosuppressive therapy, including corticosteroids, rituximab, and cyclophosphamide, remains a cornerstone of treatment, but the choice and dosage of drugs must be carefully balanced to avoid severe side effects. Comorbidity management, regular monitoring of kidney function, and a patient-centered approach are crucial for improving outcomes and quality of life. A multidisciplinary team can provide comprehensive care, addressing all aspects of the patient’s health. Supportive care, the role of kidney biopsy, and the balance between immunosuppressive therapy and the risk of complications are emphasized. Collaborative, individualized care approaches are recommended to improve outcomes and quality of life for elderly patients with immune-mediated kidney diseases. Future research should focus on including older patients in clinical trials to establish robust, age-specific guidelines.

## Introduction

The classical definition sets the elderly as the time of life over 65 years from birth. However, in the literature, every attempt to classify elderly patients tends to be subjective since every individual has a different adaptation to aging. Acquired risk factors (e.g., exposure to risk factors, past work activity) and dissimilar tolerance against its consequences (e.g., genetics) are often sufficient to explain a different phenotype between equal-in-age individuals [1]. A further rapidly escalating subclass is represented by ‘very elderly’ (>80 years), where differences between individuals are often much more substantial [2].

In the last twenty years, social health programs have dramatically improved the overall survival of these individuals, increasing the demand for a higher quality of life. However, those ≥75 years are often represented by fragile individuals who struggle to tolerate appropriate treatment of most morbid conditions. In geriatrics, the concepts of ‘pre-old age’ and ‘old age’ have been introduced for those aged 65–74 years and ≥75 years, respectively. Unfortunately, this stratification alone is insufficient as a sole criterion to set diagnostic or therapeutical recommendations, especially for those ≥75 years, since in the literature, there is a concerning lack of evidence on how to manage most diseases in this group of patients [3].

As nephrologists, we are frequently called to action when these kidney disorders appear in older adults. However, deep-rooted preconceptions often delay diagnosis (e.g., the safety of kidney biopsy in the elderly) or treatment, potentially exposing them to undertreatment.

In older adults, glomerular diseases directly impact kidney function and exacerbate preexisting cardiovascular, metabolic, and functional vulnerabilities; the pathogenetic insult can be direct (e.g., ANCA-associated vasculitis) or indirect (e.g., nephrotic syndrome). These patients are at increased risk of cardiovascular disease *per se* because of aging, driven by hypertension and atherosclerosis, as well as metabolic disturbances, including sarcopenia and malnutrition. When existing, these conditions define frailty, a clinical syndrome linked to aging, marked by reduced physiological reserves in response to stress. It has a 4% to 14% prevalence in several European countries, leading to increased hospitalization, dependency, and mortality [4]. Comprehensive management should, therefore, address these interconnected factors to optimize patient outcomes.

A comprehensive, systematic assessment is crucial, involving evaluating comorbidities, functional and cognitive status, social and nutritional factors, and the impact of polypharmacy, even if modulated [4]. The Fried scale can measure the frailty in the overall population [5]. Frailty is often further enhanced by the treatment’s side effects, which can lead to death.

### Clinical workup

In older adults, kidney disease is more elusive than in other age groups because (1) a clear definition of functional decline of the kidney and morbid state is not offered in the literature as the actual laboratory or noninvasive tests are not sufficiently sensitive or specific, and (2) most clinical trials exclude elderly patients.

**Figure 1.**
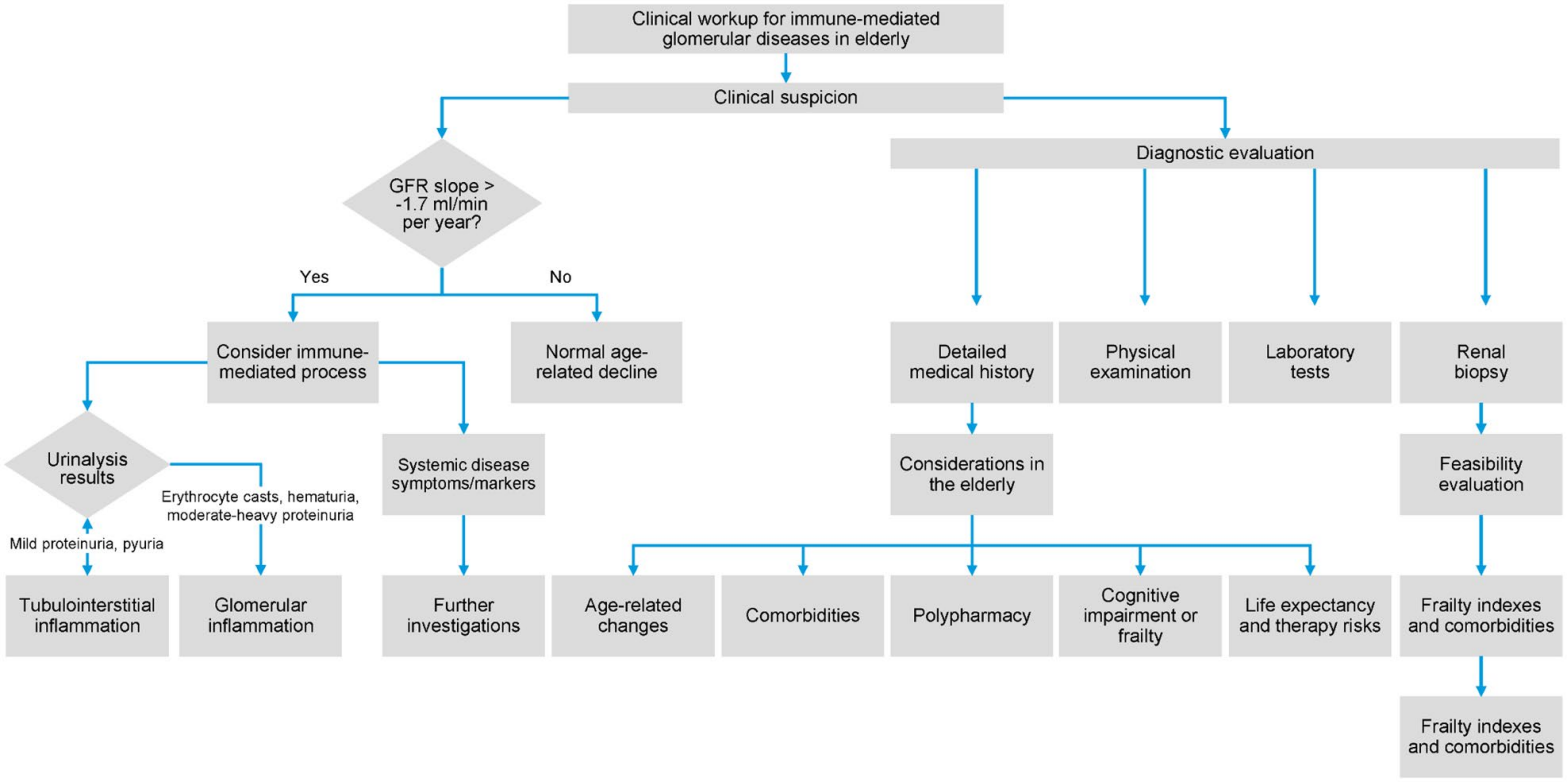
Flowchart for the clinical workup of immune-mediated glomerular diseases in the elderly.

Clinical suspicion of an ongoing immune-mediated process arises from a few key considerations (Figure 1). First, given that a mild, time-related kidney function decline is expected in healthy individuals, the glomerular filtration rate (GFR) slope should not exceed −1.7 mL/min per year in >70-year-old [6]. Second, urinalysis must be investigated to rule out most inflammatory renal diseases; in fact, mild proteinuria and active urine sediment (leukocyturia, inflammatory casts, hematuria) are observed in tubulointerstitial inflammation while, in contrast, erythrocyte casts, microscopic hematuria with acanthocytes, and moderate to heavy proteinuria are specific indicators of glomerular inflammation. Third, symptoms or biochemical markers of systemic diseases (e.g., paraproteins, autoimmunity, microbiology) with kidney involvement must prompt additional investigations when suspecting a secondary involvement.

The clinical workup for immune-mediated glomerular diseases in the elderly requires a comprehensive approach, considering the unique challenges and complexities in this population.

#### Diagnostic evaluation

- Detailed medical history, including comorbidities, medications, and potential exposures to nephrotoxins or allergens.
- Physical examination, with emphasis on signs of systemic involvement (e.g., rash, arthritis, serositis).
- Laboratory tests:
  - Urinalysis (proteinuria, hematuria, casts)
  - Serum creatinine and estimated glomerular filtration rate (eGFR)
  - Serological tests (ANA, ANCA, anti-GBM antibodies, complement levels, cryoglobulins)
  - Serum and urine protein electrophoresis (for monoclonal gammopathies)
- Renal biopsy is often necessary for definitive diagnosis and to guide treatment decisions, especially in cases of rapidly progressive glomerulonephritis or when the diagnosis is uncertain.

#### Considerations in the elderly

- Age-related physiological changes, such as decreased renal mass and GFR, can complicate the interpretation of laboratory findings.
- Comorbidities like hypertension, diabetes, and atherosclerosis are common and can contribute to or mimic glomerular disease.
- Polypharmacy and potential drug interactions or nephrotoxicity should be evaluated.
- Cognitive impairment or frailty may impact treatment adherence and decision-making.
- Life expectancy and potential risks and benefits of immunosuppressive therapy should be carefully weighed.

The diagnostic workup should be tailored to the individual patient, considering their overall health status, comorbidities, and potential risks and benefits of invasive procedures or immunosuppressive therapy [7]. Close collaboration between nephrologists, rheumatologists, and other specialists is often necessary for optimal management.

If a kidney biopsy is recommended, it should be preceded by a feasibility evaluation, including frailty indexes and comorbidities. Kidney biopsy is as safe in this population as in younger individuals [8, 9].

### Treatment: the general principles

Supportive care should be offered to prevent bone disease (vitamin D supplementation, bisphosphonates), thromboembolism (if necessary, anticoagulation with vitamin K antagonists or heparin), cardiovascular events (low salt diet 2–4 g/day, appropriate LDL reduction <100 mg/dl), and glomerular hypertension (renin-angiotensin-aldosterone inhibition (RAASi)).

Once a histological diagnosis is available, the global decline of organ function can challenge our therapeutic program. In fact, in frail patients, the risks of immunosuppression may outweigh the benefits. Any therapeutic regimen should be tailored by accounting for kidney and liver function (e.g., drug titration) and volume of distribution (e.g., calcineurin inhibitors (CNI)). Moreover, preventing opportunistic infections (e.g., Pneumocystis jirovecii) has a prominent role in our patients. Of note, sodium-glucose cotransporter 2 inhibitors (SGLT2i) have been introduced in practice in proteinuric patients with nondiabetic kidney disease. Still, their role in patients with active glomerular diseases on immunosuppressive therapy is unclear regarding outcomes and side effects [10].

### Treatment: the specific approach

#### Minimal change disease (MCD)

MCD is a primary podocytopathy clinically expressed with moderate to severe nephrotic syndrome. It is infrequent in elderly people (2.2% of diagnoses) [11]. At the time of this review, no randomized clinical trials involved this population; thus, high-quality evidence does not support a specific treatment.

Management is challenging in adults with severe presentations since (1) MCD is often associated with acute tubular necrosis due to fluid unbalance and high-dose diuretics needed to counteract the edema [12]; we do not have a case series that reports AKI incidence in the elderly, but it is plausible that it manifests in 20%-25% as in adult patients [13]; (2) the time to achieve remission is longer compared with younger patients (hazard ratio 0.53 (CI 95% 0.32–0.88)), while relapse rates are lower (HR 0.39 (0.21–0.74)) in more recent case series [12]; (3) mortality rates are increased compared to younger patients (in dated series, the overall survival of > 60-year-old at onset is only 50% at ten years) [14].

Glucocorticoids are widely considered the first line of treatment, as inferred from clinical trials involving pediatrics and younger adults (see Table 1). However, in the elderly, conventional doses are commonly not tolerated (salt retention, myalgias, hyperglycemia, insomnia, psychiatric disorders, infections, etc.), especially while on a high dose and long-term regimens; in particular, compared with younger patients, elderly patients with MCD have 13.3% more hospitalizations due to infection for >2 weeks, cataract (15.6%), and diabetes (26.7%) [14].

**Table 1.** Treatment recommendations for glomerulonephritis in elderly patients.

Kidney disease	Treatment
General indications − 1 (If no specific contraindication)	All patients: Low salt diet (2–4 g/day) Blood pressure control: use ACEi/ARBs and loop diuretics Dyslipidemia: statins, fibrates, omega-3 fatty acids Appropriate vaccinations RAASi (ACEis or ARBs) Consider associating an SGLT2i Promote aerobic exercise
General indications − 2 (immunosuppression)	Prevent infections according to the chosen immunosuppression (e.g., PJP prophylaxis) Drugs should be fitted for age, GFR (e.g., reduce CYC to 50% in >70-year-old), blood levels (e.g., CSA), and side effects If glucocorticoids are included: Prevent bone loss: consider 25OH-vitamin D and oral calcium supplementation Prevent gastric bleeding if preexisting risk is present Monitor blood sugar Follow a dose marker (e.g., CD20+ in RTX, ANC 1500/mm3 in CYC)
Minimal Change Disease	If high-dose glucocorticoids (GC) are well tolerated: Prednisone 60 mg/24h (1 mg/kg/24 hr) for the first week, decreasing to 45 mg (0.75 mg/kg)/24h in the second week, and after that for a further four weeks in 8–20 weeks [15] If high-dose GCs are not tolerated Consider CSA or TAC in monotherapy or add CYC to a tolerated dose of GCs [16] Steroid-dependent or frequent relapsers: consider RTX (CD19 target: 0%) or MMF (1000 mg/12h) plus low-dose prednisone (10 mg/day) [17, 18]. Steroid-resistant: consider CSA or TAC in monotherapy or CYC with a tolerated dose of steroids [15]
Focal and Segmental Glomerulosclerosis, primary	As per MCD [15, 19]. There are no available RCTs for primary FSGS, including ≥ 75-year-olds.
Membranous Nephropathy - anti PLA2R - anti THSD7A - other antigens	Frail older adults: consider supportive therapy only Fit older adults: Treat if: Absence of spontaneous remission after six months on RAAS inhibitors High anti-PLA2R titer at presentation (≥200 RU/ml) Age-recommended cancer screening is negative Treat immediately if: High risk of progression (GFR <45 mL/min, increase in serum creatinine by more than 25% in 6 months) Failure to control symptoms Immunosuppressive therapy: RTX 1000 mg every two weeks in a month [20] Repeat another infusion by 6 months if proteinuria does not reach the baseline Consider CYC-based regimens after RTX failure (inferior safety profile) [21] 1.5 mg/kg/day for 12 months (Nijmegen schedule) or 2.5 mg/kg/day on months 2, 4 and 6 (Ponticelli’s schedule), with a strict control of leukocyte count (Ponticelli’s: if leukocytes <5000/mm3 or platelets <100.000/mm3, discontinue CYC for remainder of cycle. Nijmegen’s: if leukocytes <5000/mm3 or platelets <100/mm3, dose should be halved; if leukocytes <3000/mm3 or platelets <75/mm3, discontinue CYC). Consider CSA or TAC if alkylating agents are contraindicated [20]
ANCA associated vasculitis -Microscopic polyangiitis (MPA) -Granulomatosis with polyangiitis (GPA)	If there is evidence of organ involvement, treat Induction**:** Plasmapheresis: its role is controversial in kidney and overall survival. In general, it is not recommended [22–24] I.V. glucocorticoids (MP, three pulses with a cumulative dose of 1–3 g), followed by prednisone full dose the first week (60 mg/day), then 30 mg/day and taper to 7.5 mg/day by three months and 5 mg/day by six months [22] RTX-based regimens PEXIVAS - RTX 375 mg/m2 per week for four weeks [22] CYC-based regimen I.V. (15 mg/kg/pulse) or oral (2 mg/kg/day) for 3–6 months [22] RTX + CYC regimen RITUXVAS - RTX 375 mg/m^2^ per week for four weeks plus two I.V. CYC (dose adjusted to 7.5 mg/kg) [24] Other regimens based on different combinations of the above [25, 26] Maintenance**:** RTX regimens (if RTX induced), fixed or tailored [23] MMF 2–3 g/day alone +/- low dose GCs [15] AZA at a target dose of 2 mg/kg/day +/- low-dose GCs [22–24]
Anti-GBM disease	Immediate Treatment: Initiate high-dose glucocorticoids: three pulses of i.v. MP 15 to 30 mg/kg to a maximum dose of 1000 mg, followed by prednisone 1 mg/kg per day to a maximum of 60 to 80 mg/day CYC: start 2 mg/kg per day orally, reducing the dose by 25% in older and frail patients. Plasmapheresis: at least four-liter exchanges for two to three weeks (a mean of 9 exchanges) Replace with fresh frozen plasma in cases of alveolar hemorrhage or coagulation disorders or recent kidney biopsy, otherwise use albumin. Individualized Immunosuppression, adjusting based on age, renal function, and frailty (target lymphocyte count <700/mm³). Monitor Anti-GBM Titers: If antibodies persist beyond 14 days, consider prolonged immunosuppression or rituximab. Otherwise, taper prednisone and cyclophosphamide and reduce the frequency of plasmapheresis.
IgA nephropathy and IgA vasculitis	Exclude a Staph infection Frail older adults: supportive therapy only Fit older adults: Review the kidney biopsy: No endocapillary or crescentic proliferation: ACEi or ARBs Endocapillary proliferation: consider steroids Eventually, add MMF as a steroid-sparing agent Crescentic proliferation: consider CYC + steroids
Infection-related Glomerulonephritis	Treat the infection Supportive therapy Immunosuppressive drugs and glucocorticoids are **not** recommended [27]
Post-Infectious Glomerulonephritis	Supportive therapy Immunosuppressive drugs and glucocorticoids are **not** recommended (some expert opinions still recommend alkylating agents if ≥30% crescents on kidney biopsy) [27]
Lupus Nephritis - Class III-IV - Class V	**Always treat if**: (1) fit patient; (2) active disease (e.g., active urine sediment, nephrotic syndrome); (3) negative cancer screening **Induction**: Glucocorticoids: MP i.v. 250–500 mg daily for three days Then: Prednisone 60 mg/day tapered every two weeks by 10 mg/day until 40 mg/day, then taper by 5 mg/day until 10 mg/day is reached (a 5 mg/day dose is frequently allowed for long term). Plus (choose): MMF 2–3 g/day (depending on tolerance) Consider the association with a calcineurin inhibitor (tacrolimus or voclosporin) or belimumab to reduce toxicity oral CYC (adjust for kidney function), not over 100 mg/day Consider the association with belimumab to reduce toxicity **Maintenance** MMF 2 g/day, tailored to clinical response and side effects + low dose prednisone
C3 glomerulopathies	Older adults are likely to have a monoclonal protein acting as a C3 convertase stabilizer or having anti-inhibitor activity Perform a full complement investigation [28] Frail older adults: supportive therapy only Fit older adults: Evaluate the kidney biopsy to stratify the progression risk (e.g., crescents, severe endocapillary proliferation) and find histological clues (e.g., monoclonal restriction). If potentially progressive: Polyclonal antibodies deserve general immunosuppressive therapy e.g., MMF as per lupus nephritis Monoclonal antibodies should be addressed as an MGRS

ACEi: angiotensin-converting enzyme inhibitor; ANC: absolute neutrophil count; ARB: angiotensin receptor blocker; AZA: azathioprine; CSA: cyclosporine A; CYC: cyclophosphamide; GC: glucocorticoid; GFR: glomerular filtration rate; I.V.: intravenous; MGRS: monoclonal gammopathy of renal significance; MMF: mycophenolate mofetil; MP: methylprednisolone; PJP: Pneumocystis jirovecii pneumonia; RAASi: renin-angiotensin-aldosterone system inhibitor; RCT: randomized controlled trial; RTX: rituximab; SGLT2i: sodium-glucose cotransporter-2 inhibitor; TAC: tacrolimus.

If not tolerated, it is a common practice to consider a steroid-sparing strategy. CNIs, particularly tacrolimus (TAC), have been used as monotherapy in a cohort of older patients, with results similar to glucocorticoid-based regimens regarding kidney response, rate of relapse, and time to relapse [14, 16, 29].

In relapsing MCD, cyclophosphamide (CYC) centered regimens offered more sustained long-term remissions than corticosteroid-based regimens and a remission rate of 69% within 16 weeks in patients over 60, with a similar safety profile [14]. Rituximab is an alternative in frequent relapsers or steroid-dependent patients, considering its favorable safety profile, although no data are available from clinical trials in elderly patients [17]. Mycophenolate mofetil (MMF) has a theoretical basis and provides effects comparable to CNIs in young relapsers, but its role in older individuals is unknown [18].

#### Focal and segmental glomerulosclerosis (FSGS), primary

Primary FSGS is a podocytopathy representing 9.1% of kidney biopsies performed in patients over 75 years [11]. In older patients, secondary forms of FSGS must be carefully ruled out (adaptive, scarring, iatrogenic, and rarely genetic) since these forms are not an expression of an immune process; for this reason, electron microscopy is pivotal in documenting extensive foot process effacement >80% [30]. On the other hand, secondary FSGS only deserves anti-progression measures.

Primary FSGS shares several analogies with MCD immunosuppressive protocols. However, there are no clinical trials that include elderly cohorts. First-line therapy is, as in MCD, high-dose corticosteroids (see Table 1). The most significant experience published dates back to 1994, which used a high-dose, alternate-day prednisone regimen proposed in patients >60 years of age, obtaining 44% of complete remissions after 3–5 months of treatment [19]. However, there is broad agreement that steroid-sparing agents should be considered. Other recommendations for steroid intolerance and frequent relapsers are in line with MCD disease.

#### Membranous nephropathy

It is a histological pattern characterized by granular sub-epithelial deposits of polyclonal immunoglobulins and C3, clinically expressed as moderate to heavy proteinuria reaching nephrotic syndrome [31]. Most cases (70%) are constituted by immune deposits of IgG4 directed against the M-type receptor of phospholipase A2 (PLA2R); the remaining cases represent other antigens whose clinical significance is under assessment [15].

In general, membranous nephropathy should be treated when there is evidence of a trend toward end-stage kidney disease (ESKD), namely progressive renal failure, highly symptomatic nephrotic syndrome, and inadequate relief of proteinuria to renin-angiotensin inhibitors (less than 50% after six months) [32]. Otherwise, supportive therapy is recommended by recent KDIGO 2021 guidelines since spontaneous complete remission of proteinuria can occur in a third of affected patients at five years [32]. Moreover, spontaneous partial remission was observed in 42% of patients after five years of follow-up in an Italian cohort involving patients >70 years with a low risk of developing ESKD [33]. On the other hand, a Toronto series described a progressive risk of observing severe fibrosis and tubular atrophy, vascular sclerosis, and FSGS in older patients, with lower remission rates in those with more severe chronicity indexes (e.g., remission without immunosuppression: 84% with a score 0 in atrophy or fibrosis, 33% in score 3+; 72% with a score 0 in vascular sclerosis, 43% in score 3+) [34].

Although there is uncertainty in defining the prognosis due to contradicting experiences, primarily due to unmodifiable factors such as age, unfortunately, no clinical trials on membranous nephropathy involve patients ≥75 years old; thus, the role of immunosuppression in these patients is not determined. Is the long-term poor prognosis and clinical response driven by age-related lesions or by the immunologic process itself?

The therapeutic approach is similarly challenging. In younger patients, most authors agree to start immunosuppression immediately in those with severe nephrotic syndrome and renal failure after three to six months of supportive measures [34, 35]. However, in antiPLA2R-positive patients, the titer of circulating antibodies that define the immunological activity is predictive of clinical remission and guides the treatment [35]. If the antibody titer is low, spontaneous or induced immunological remission is more likely, preceding the remission of nephrotic syndrome; on the other hand, if the titer is high (212.9; IQR, 116.5–352.6 RU/mL but KDIGO 2021 accepts ≥200 RU/mL), thus in the absence of immunological remission, the clinical remission is unlikely to be achieved [36]. In the latter event, treatment could be considered in fit patients or those where the risk of immunosuppression is inferior compared to their clinical risk of developing complications of nephrotic syndrome or worsening quality of life.

In our practice, anti-CD20 treatment (rituximab) is the first-line treatment because of its favorable safety profile compared to other agents (e.g., cyclophosphamide) and fewer relapses according to the MENTOR trial, the latter typical of calcineurin inhibitors (see Table 1) [20, 21]. The only evidence of the role of RTX in our age target emerged from an observational study from the Mayo Clinic in a subset of high-risk patients (baseline proteinuria 11.9 g/24h), with good outcomes (16/18 reached clinical remission) after 24 months of follow-up using a 375 mg/m2 x4 schedule and retreated with a single infusion at month six regardless their response [37]. In case of failure, some authors recommended CYC-based regimens, oral or pulse-based, with strict control of leukocyte count (Table 1) [38].

#### ANCA-associated vasculitis (AAV)

AAVs are a group of small vessel vasculitis that includes microscopic polyangiitis (MPA), granulomatosis with polyangiitis (GPA), and eosinophilic granulomatosis with polyangiitis. MPA and GPA are relatively frequent in older people compared to other decades of life. MPA and GPA mainly involve the lungs and kidneys, leading to rapidly progressive kidney failure, hemoptysis, and respiratory failure. Necrotizing, pauci-immune glomerulonephritis dominates kidney histology with rapid or indolent evolution [39].

As observed in the pretreatment era, mortality is elevated if the active disease is left untreated while, despite specific treatment, increasing the odds of infections (standardized mortality ratio, SMR, 13.9), malignancy (SMR 2.7), and cardiovascular disease (SMR 2.3) [40]. The latter was significantly higher in MPO-ANCA patients (HR 4.98). For these reasons, an immunosuppressive regimen with the best safety profile should be proposed, particularly avoiding opportunistic infections.

For induction, we adopt rituximab-based regimens over cyclophosphamide-based regimens because of their favorable side effects profile and similar response rate [41]. For inducing remission, RTX-based regimens are founded on fixed doses (375 mg/m2 x4 weeks, or 1 gram every 15 days for a total of 2 infusions), which are equivalent in terms of side effects and risk of relapse [22–24]. On the other hand, induction with CYC-based regimens must consider a dose adjustment (50% in elderly patients) and exposure within 3 to 6 months, followed by AZA or MMF in the long term [22]. Other regimens included a small part of elderly patients with different therapeutic strategies based on the same molecules [25, 26]. Generally, most authors adopt pulses of methylprednisolone with a cumulative dose of 1–3 g [15].

Despite the number of trials, only two clinical trials on AAV included elderly patients: the ‘Plasma Exchange and Glucocorticoid Dosing in the Treatment of ANCA-Associated Vasculitis’ (PEXIVAS) and the ‘Avacopan Development in Vasculitis to Obtain Corticosteroid Elimination’ (ADVOCATE) [22, 42].

The PEXIVAS trial, involving 704 patients, assessed the efficacy of plasma exchange and reduced-dose glucocorticoids in severe ANCA-associated vasculitis. The study found that (1) the composite outcome of death from any cause or end-stage kidney disease occurred in 28% of patients in the plasma exchange group compared to 31% in the control group, showing no significant benefit (hazard ratio 0.86, 95% CI 0.65–1.13) and (2) the reduced-dose glucocorticoid regimen was non-inferior to the standard-dose regimen, with 28% and 26% of patients, respectively, experiencing the primary outcome (absolute risk difference 2.3%, 90% CI −3.4% to 8.0%), with fewer severe side effects [22]. Low-dose corticosteroids were postulated in AAV and compared against high doses in another publication, confirming this finding [43].

Despite these findings, some authors still consider plasma exchange a valuable therapeutic option for patients with GPA or MPA under specific conditions. PLEX may be of help in patients with severe kidney disease, defined by a serum creatinine level >4.0 mg/dL or those requiring dialysis, as well as in cases of pulmonary hemorrhage, where it may prevent life-threatening complications. Additionally, PLEX is advised for patients who are concurrently positive for anti-glomerular basement membrane (anti-GBM) antibodies, as this treatment aligns with the management approach for anti-GBM disease.

In 2021, the selective inhibitor of the C5a receptor avacopan has emerged as a promising therapeutic agent for patients with ANCA-associated vasculitis. The ADVOCATE study, a phase 3 randomized controlled trial, evaluated the efficacy and safety of avacopan (30 mg twice a day) compared to prednisone in combination with standard immunosuppressive therapy, including a small cohort of elderly patients. The study demonstrated that avacopan was non-inferior to prednisone in achieving remission at 26 weeks (72.3% vs. 70.1%, estimated common difference, 3.4 percentage points; 95% CI, −6.0 to 12.8; *p* < 0.001 for noninferiority; *p* = 0.24 for superiority) and superior in maintaining sustained remission at 52 weeks (65.7% vs. 54.9%, estimated common difference, 12.5 percentage points; 95% CI, 2.6 to 22.3; *p* < 0.001 for noninferiority; *p* = 0.007 for superiority). The latter outcome is a matter of debate since prednisone was stopped at 26 weeks as per the trial protocol. Additionally, avacopan was associated with fewer toxicities, highlighting its potential to reduce the burden of long-term steroid use in this patient population [42].

#### Anti-GBM disease

Anti-GBM disease is caused by circulating antibodies against the NC1 domain of the alpha-3 chain of type IV collagen. The clinical presentation is a reno-pulmonary syndrome with an incidence of alveolar hemorrhage of 40% and severe kidney failure requiring replacement therapy in 63% [44]. This targeted pathogenesis is justified by the expression of type IV collagen in the alveolar and glomerular basal membrane, while other sites (e.g., cornea) are exempted because they are not directly exposed to circulating antibodies. The age of onset has two peaks between the second to third and sixth to seventh decades (13% over 75 years) [44]. The classic histological pattern is a diffuse crescentic glomerulonephritis with a typical linear positivity for IgG on GBM.

The standards of care are high-dose glucocorticoids plus cyclophosphamide and plasmapheresis. Unfortunately, in the literature there is only a randomized clinical trial that did not include patients over 75 years old [45]. However, in the general population, immunosuppression dramatically changes the course of the disease regarding renal and overall survival; we recommend starting immunosuppression and plasmapheresis as soon as the diagnosis is made (see Table 1). Considering the high frailty index of these patients with active disease, immunosuppression should be carefully modulated according to renal function, age, and side effects (consider a target lymphocyte count of <700/mm3). In contrast, plasma exchange should be combined with fresh frozen plasma in case of alveolar hemorrhage or evidence of coagulation disorders. Typically, treatment should be tailored according to the anti-GBM titer; in most cases, antibodies disappear from plasma after 14 days of treatment. However, exceptions are observed and deserve prolonged immunosuppression or a change of induction strategy (e.g., rituximab) [46].

#### IgA vasculitis

IgA vasculitis (IgAV), formerly known as Henoch-Schönlein purpura, is a leukocytoclastic, immune complex-mediated, small-vessel vasculitis with IgA1-dominant immune deposits. It is the most common systemic vasculitis in childhood, with an annual incidence of 3–26 per 100,000 children and a mean age of 6 years. In adults, the median incidence of IgAV is 1.3 cases/100,000 adults/year, with a median age of disease manifestation of 50 years, although observed >75 years. This cohort has a substantial risk of kidney failure and of developing hypertension, vasculopathy, and cardiovascular death [47, 48].

Unfortunately, no controlled studies specifically target patients over 75 with IgAV. In younger adults, controlled data are scarce, with most evidence from case series suggesting a potential benefit from immunosuppressive therapies. A retrospective study from China included ninety-five adults with IgAV and kidney involvement, comparing MMF to glucocorticoids and RAAS inhibitors. Proteinuria was significantly lower in the MMF and steroid groups than controls [49]. Additionally, rituximab and other anti–B cell therapies are showing promise in refractory or relapsing cases. In a case series of 22 adults, 90% achieved remission with rituximab, although 35% relapsed, with a significant reduction in proteinuria and steroid use [50].

#### Infection-related glomerulonephritis

The elderly are at increased risk for infection-related glomerulonephritis due to a decline in immune function, making them more susceptible to infections [51]. However, the true incidence of this condition in the elderly remains unclear, partly due to challenges in patient cooperation during diagnostic procedures, as well as the confounding presence of hematuria from catheterization, which can obscure the diagnosis and lead clinicians to mistakenly attribute the findings to more common conditions like acute tubular necrosis. Additionally, the lack of a standardized definition for infection-related glomerulonephritis in this age group further complicates accurate identification and reporting [27].

Infection-related glomerulonephritis is defined by (1) evidence of an ongoing infection and (2) a concurrent nephritic syndrome or a urinalysis suggestive of glomerular inflammation [27]. β-hemolytic Streptococcus is frequently involved, especially causing infection of the upper respiratory tract (e.g., pharyngitis, sinusitis, otitis media) and deep tissues (e.g., cellulitis). Kidney involvement occurs after a few days up to 2–3 weeks from the onset of infection, giving a global, diffuse, proliferative glomerulonephritis, rich in mesangial, subendothelial, and subepithelial deposits (the latter called ‘humps’). The inflammatory response is primarily driven by neutrophils and lymphocytes, which migrate *via* diapedesis from the capillaries into the mesangial and subendothelial areas. This process is mainly triggered by bacterial debris and complement (C3) deposits, with a lesser contribution from immunoglobulins (IgG). These deposits precipitate in the glomerular structures, resulting in the characteristic ‘starry sky’ pattern in immunofluorescence [52].

Antibiotic therapy is the ‘gold standard’ of treatment (Table 1). Immunosuppression is contraindicated, although its timing and use when severe crescentic glomerulonephritis is observed are debated. Unfortunately, recommendations are based only on expert opinions [27].

#### Post-infectious glomerulonephritis

Post-infectious glomerulonephritis is defined by: (1) evidence of recent infection, no longer in place at the time of diagnosis; (2) latency, from 1 to 4 weeks; (3) kidney involvement (nephritic syndrome often with nephrotic syndrome and rapidly progressive glomerulonephritis). This entity is typical of childhood and, in adults, has a likely underestimated incidence of 0.4 cases/100.000/year [53]. Causes span from skin, urinary, and respiratory infections [54]. The most plausible pathogenic mechanism is autoimmune, after molecular mimicry with bacterial antigens. A kidney biopsy should be performed for diagnostic confirmation, showing a pattern similar to infection-related glomerulonephritis. The therapeutic approach is mainly supportive since inflammation tends to remit. However, some authors recommend the use of immunosuppressive agents if extra-capillary proliferation is observed in ≥30% of glomeruli (Table 1) [27].

#### Other glomerulonephritides

In adults, Lupus nephritis, IgA nephropathy, and C3 glomerulopathies are other rare forms of glomerulonephritis.

Although infrequent, late-onset lupus nephritis shows a clinical picture identical to that of younger individuals. Evaluation should focus on (1) the exclusion of cancer and a drug review and (2) the evidence of clinical/histological activity. Excluding those patients at high risk of side effects for whom the treatment should be limited and individualized, in our practice, the therapeutic approach is almost identical to that in younger individuals [55, 56].

IgAN is associated with a more rapid and aggressive presentation in older patients. There are no data on the treatment of this cohort of patients. Realistically, patients with evidence of benign course should be approached with RAAS inhibition, while rapidly progressing patients must be evaluated for immunosuppression based on histological evidence of active lesions; the latter group needs an accurate differential diagnosis with Staphylococcal-induced IgA nephropathy, where immunosuppression is contraindicated (see infection-related and post-infectious GNs).

C3 glomerulopathies with late-onset are mainly mediated by paraproteins or are the expression of autoimmune or infection-related GNs [28]. In these cases, the therapeutic approach must be directed against the offending agent.

### Personalized medicine approach

The application of personalized medicine in elderly patients with immune-mediated nephropathies requires tailoring interventions to account for the unique physiological, pharmacological, and genetic factors of older adults. In this population, age-related changes in drug metabolism, polypharmacy, and frailty can significantly affect both treatment efficacy and tolerability. Recent studies emphasize the role of pharmacogenomics in optimizing immunosuppressive therapies in older patients, particularly by adjusting drug dosages to reduce adverse events, such as infections and cardiovascular complications, which are more prevalent in this demographic. For instance, lower doses of rituximab were effective in older adults with membranous nephropathy, minimizing the risk of drug toxicity without compromising efficacy [57]. Moreover, biomarkers like anti-PLA2R antibodies have proven useful in predicting treatment responses in older adults with membranous nephropathy, allowing for more precise immunosuppressive regimens that balance efficacy with safety. By integrating advanced diagnostic tools, such as genetic testing and biomarker analysis, clinicians can better predict responses to therapies and minimize adverse effects in elderly patients. The ultimate goal is to enhance the quality of life and functional status in this vulnerable group, reducing the burden of comorbidities and medication side effects.

### Holistic approaches

Holistic approaches to managing immune-mediated nephropathies in the elderly must go beyond general wellness strategies to address the specific physical, emotional, and social challenges faced by this population. Older adults often have complex comorbidities, including cardiovascular disease, diabetes, and cognitive impairment, which complicate the management of nephropathies. The integration of nephrologists, geriatricians, and mental health professionals into care teams has shown promising outcomes in terms of reducing hospitalizations and improving patient-reported quality of life. For example, a multidisciplinary model trial demonstrated that older patients with chronic kidney disease who received coordinated care from a geriatric-nephrology team experienced a 25% reduction in the rate of functional decline [58]. Additionally, lifestyle interventions tailored to elderly patients, such as low-intensity physical activity programs and renal-specific dietary modifications, have been shown to slow disease progression while improving patient engagement. These interventions are particularly important in preventing frailty, which is strongly associated with worse outcomes in elderly nephrology patients. Holistic care for elderly individuals should also emphasize psychosocial support, addressing issues such as loneliness, depression, and the psychological impact of chronic disease, which are more prevalent in this age group. Such a comprehensive, patient-centered approach ensures that the elderly receive care that respects their unique vulnerabilities while promoting autonomy and quality of life.

### Future studies

Future studies should focus on including elderly patients in clinical trials to develop robust, age-specific guidelines for managing immune-mediated nephropathies (Table 2). These studies should address the unique physiological changes, comorbidities, and treatment responses in this population. Understanding the long-term outcomes and potential side effects of immunosuppressive therapies in the elderly will help refine treatment strategies. Additionally, research on biomarkers and noninvasive diagnostic tools tailored for the elderly can improve early detection and monitoring, ultimately enhancing the quality of care and life expectancy for these patients.

**Table 2.** Areas for future studies in elderly immune-mediated nephropathies.

Study Focus	Rationale
Inclusion of Elderly in Clinical Trials	Develop age-specific treatment guidelines; understand unique responses and side effects.
Long-term Outcomes of Immunosuppressive Therapies	Evaluate safety and efficacy of treatments; refine therapeutic strategies for elderly patients.
Biomarkers for Early Detection	Improve early diagnosis and monitoring; tailor diagnostic tools to the elderly population.
Noninvasive Diagnostic Tools	Reduce risks associated with invasive procedures; enhance patient comfort and compliance.
Management of Comorbidities	Address the impact of multiple health conditions on treatment outcomes; improve overall health.
Patient-Centered Care Approaches	Tailor treatment plans to individual health profiles and preferences; enhance quality of life.
Prevention of Opportunistic Infections	Develop strategies to minimize infection risks; ensure safe immunosuppression practices.
Pharmacokinetics and Pharmacodynamics in Elderly	Understand drug metabolism and effects in the elderly; optimize dosing regimens.
Multidisciplinary Care Models	Promote collaborative care approaches; integrate services from various specialties for holistic care.

## Conclusions

In light of the current evidence, elderly patients with immune-mediated nephropathies deserve an age-independent diagnostic, prognostic, and therapeutic approach; however, each case must be evaluated according to the existing comorbidities, respecting the risk/benefit ratio. Our review showed that it is essential to include older patients in risk profiling studies and determine the efficacy of new therapies since this group is rapidly growing in most countries.
